# Safety and Efficacy Associated With a Family-Centered Procedural Sedation Protocol for Children With Autism Spectrum Disorder or Developmental Delay

**DOI:** 10.1001/jamanetworkopen.2023.15974

**Published:** 2023-05-30

**Authors:** Cyril Sahyoun, Baruch Krauss, Martina Bevacqua, Artiom Antonsen, Laurent Jardinier, Egidio Barbi

**Affiliations:** 1 Division of Pediatric Emergency Medicine, Children’s Hospital of Geneva, Geneva University Hospitals, Geneva, Switzerland; 2 Division of Emergency Medicine, Boston Children’s Hospital, Department of Pediatrics, Harvard Medical School, Boston, Massachusetts; 3 Divisions of General Pediatrics and Pediatric Subspecialties, Children’s Hospital of Geneva, Geneva University Hospitals, Geneva, Switzerland; 4 Department of Pediatrics, Institute for Maternal and Child Health, IRCCS Burlo Garofolo, Trieste, Italy; 5 University of Trieste, Trieste, Italy

## Abstract

This case series describes a family-centered procedural sedation protocol including home desensitization to intranasal drug delivery, environmental modification, and intranasal dexmedetomidine combined with nitrous oxide for children with autism spectrum disorder or developmental delay.

## Introduction

Routine health care, whether physical examinations, blood sampling, vaccination, or dental care, is challenging and often traumatic for children with autism spectrum disorder (ASD) and children with developmental delay (DD), often requiring physical restraint or being indefinitely postponed. Behavioral interventions are critical for effective treatment of these children; however, in many children who have had traumatic medical experiences, these interventions may not be successful.^1^ Pharmacologic treatment with oral sedatives may be insufficient to perform common minor procedures, while the intramuscular route, requiring physical restraint for administration, can deepen the fear and lack of trust these children have toward the health care system.^2^ We describe a consecutive case series using a family-centered integrated behavioral and sedation protocol for common medical procedures in these children.

## Methods

This case series was approved by the Swiss Association of Ethics Committees. Informed consent was obtained from the children's parents or legal guardians. This study is reporting following the reporting guideline for case series. We developed a procedural sedation protocol for children ages 2 to 16 years with ASD or DD described by parents as previously unapproachable for routine immunization and venipuncture due to extreme agitation. After discussion with the patient’s referring pediatrician or pediatric subspecialist and with the parents, children were enrolled for participation in the protocol. Our hypothesis was that an integrated behavioral-pharmacologic protocol is safe and effective in such patients. The main outcome measure was the need for physical restraint.

The protocol uses home desensitization to intranasal drug delivery, environmental modification, and intranasal dexmedetomidine combined with nitrous oxide (n_2_o), as dexmedetomidine alone would not allow painful procedures to be performed comfortably. Details of the protocol are provided in the eAppendix in Supplement 1. Patient, procedure, and sedation data were collected with each sedation. Study data were collected from June 2021 to December 2022 and managed using Qualtrics software (Qualtrics). Descriptive statistics were calculated using SPSS version 29.0 (IBM). Data were analyzed from January to February 2023.

## Results

During the study period, 44 children and their parents were approached for enrollment, and 43 consented. Median (IQR) patient age was 7.2 (4.5-11.4) years, and 31 patients (72%) were male. Of 42 patients who underwent the planned procedure, 39 patients (93%) had their procedures without physical restraint. Three patients required brief restraint (1 patient by 1 person and 2 patients by 2 persons), lasting less than 30 seconds. In 1 patient, the procedure was aborted because the sedation was suboptimal. There were no intermediate or sentinel adverse events during sedation or at the 24-hour follow-up call. Patient, procedure, and sedation characteristics are detailed in the Table.

**Table.** Summary of Patient, Procedure, and Sedation Characteristics^a^

Characteristic	No. (%)
Age, median (IQR), y	7.2 (4.5-11.4)
Sex	
Male	31 (72)
Female	12 (28)
Diagnosis (patient may have ≥1)	
Autism spectrum disorder	29 (67)
Developmental delay NOS	10 (23)
Epilepsy with associated developmental delay	7 (16)
Behavioral disorder NOS	4 (10)
Associated genetic or congenital disorder	8 (19)
Complex congenital heart disease	2 (5)
Chromosome 18q22 anomaly	1 (2)
Turner syndrome	1 (2)
Creatine-synthesis deficiency	1 (2)
MEN2a syndrome	1 (2)
Moebius syndrome	1 (2)
Tuberous Sclerosis	1 (2)
Treated with protocol ≥1 time	
2 Times	7 (17)
3 Times	1 (2)
Weight for age, median (IQR), percentile	75 (31-98)
Receiving a psychotropic medication (eg, aripiprazole, risperidone, or an amphetamine)	14 (33)
Tolerated EMLA placement at home for blood draw, No./total No. with blood draw (%)	31/38 (82)
Type of procedure (n = 42)	
Blood draw	38 (88)
Electrocardiogram	22 (51)
Immunization or other IM injection	15 (35)
Dental examination	5 (12)
Abdominal or cardiac ultrasonographic imaging	3 (7)
Lumbar puncture	2 (5)
Nasofibroscopy	2 (5)
Auditory evoked potentials	2 (5)
Suture removal	2 (5)
Balloon gastrostomy button replacement	1 (2)
Optic nerve examination	1 (2)
Procedures performed during a sedation, No.	
1	11 (26)
2	15 (36)
3	12 (29)
4	3 (7)
Indication for procedure (patient may have ≥1)	
General health maintenance	29 (68)
Investigation of symptoms	13 (30)
Monitor psychotropic medication effects	11 (26)
Prior to starting a psychotropic medication	2 (5)
Sedation characteristics, mean (95% CI), min	
Time from IN dexmedetomidine administration to onset of sedation (RSS 5)	28 (15-45)
Duration of n_2_o administration	18 (5-35)
Time from IN dexmedetomidine administration to awakening (RSS 2)	106 (80-181)
Time from IN dexmedetomidine administration to readiness for discharge (modified Aldrete of 9-10)^a^	132 (93-238)
Restraint during procedure^b^	3 (7)
Procedure aborted for suboptimal sedation	1 (2)
Adverse events	
During sedation	
Intermediate^c^	0
Sentinel^c^	0
Prolonged sedation (>180 min to RSS 2)	3 (7)
Vomiting	1 (2)
Seizure not requiring intervention (in a patient known to seize multiple times a day)	1 (2)
Reported at 24-hour phone call	
Slept later than usual the night of sedation	35 (81)
Agitation	1 (2)
Parental satisfaction^d^	
4	1 (2)
5	42 (98)

Abbreviations: EMLA, eutectic mixture of local anesthetics; IM, intramuscular; IN, intranasal; n_2_o, nitrous oxide; NOS, not otherwise specified; RSS, Ramsay Sedation Scale.

^a^ Details on the RSS are provided in eTable 1 in Supplement 1 and details on the Aldrete score are provided in eTable 2 in Supplement 1.

^b^ Children requiring restraint were ages 2, 7, and 16 years.

^c^ Defined per the International Committee for the Advancement of Procedural Sedation Tracking and Reporting Outcomes of Procedural Sedation.

^d^ On a scale of 0, indicating very dissatisfied, to 5, very satisfied.

## Discussion

In this case series assessing a family-centered integrated protocol for managing children with ASD and children with DD requiring common medical procedures, 93% of the patients required no physical restraint, and procedures were successfully completed in 98% of patients. The drug regimen was perceived as well tolerated, safe, and effective.

Children with ASD are often frightened by medical situations, particularly if they have a previous traumatic experience. Behavioral approaches have been used with variable success, are often time-consuming, and require multiple visits prior to the actual procedure.^3^

Intranasal dexmedetomidine has been used effectively and safely in children with ASD for nonpainful procedures.^4^ Although intranasal drug administration could be perceived as invasive and frightening, previous home desensitization, as in our protocol, could lead to greater acceptance if performed by a parent in a safe, familiar environment. Few studies have explored the combination of n_2_o and dexmedetomidine, and to our knowledge, none have explored it in children with ASD.^5,6^

The main limitation of this study is the lack of a control group with a different behavioral and pharmacologic approach. However, this study represents proof of concept of the safety and efficacy of an integrated behavioral and pharmacologic approach of home desensitization, environmental modification, and an intranasal and inhalational pharmacologic regimen. Future studies comparing this approach with other techniques are needed.

## Notes

Supplement 1. **eAppendix.** Trial Protocol **eTable 1.** Modified Ramsay Sedation Scale **eTable 2.** Modified Aldrete Recovery Score

Supplement 2.
Data Sharing Statement
