# Supplementary material for: Safety and Efficacy Associated With a Family-Centered Procedural Sedation Protocol for Children With Autism Spectrum Disorder or Developmental Delay
Source: JAMA Netw Open. 2023 May 30;6(5):e2315974. doi: 10.1001/jamanetworkopen.2023.15974 (PMC10230313; doi:10.1001/jamanetworkopen.2023.15974)
Supplement: Supplement 1. — eAppendix. Trial Protocol eTable 1. Modified Ramsay Sedation Scale eTable 2. Modified Aldrete Recovery Score [file jamanetwopen-e2315974-s001.pdf]

Supplemental Online Content

Sahyoun C, Krauss B, Bevacqua M, Antonsen A, Jardinier L, Barbi E. Safety and efficacy associated with a family-centered procedural sedation protocol for children with autism spectrum disorder or developmental delay. *JAMA Netw Open*. 2023;6(5):e2315974. doi:10.1001/jamanetworkopen.2023.15974

eAppendix. Trial Protocol
eTable 1. Modified Ramsay Sedation Scale
eTable 2. Modified Aldrete Recovery Score

This supplemental material has been provided by the authors to give readers additional information about their work.

© 2023 Sahyoun C et al. *JAMA Network Open.*

eAppendix. Trial Protocol

Eligibility and exclusion criteria

Children two to 16 years with cognitive impairment and an American Society of Anesthesiology physical status class of 2 or less, who were described by parents as previously unapproachable and extremely agitated by routine medical procedures, requiring restraint for immunization and venipuncture, were eligible. Exclusion criteria included contraindications to the use of dexmedetomidine or nitrous oxide (N_2O).

Desensitization to intranasal drug delivery

The week prior to the scheduled procedure, parents were given detailed instructions by e-mail followed by a phone conversation with a nurse, in delivering physiologic saline into their child's nostrils two to three times-a-day using a commercial saline spray, in a familiar, quiet, and perceived-as-safe environment for the child.

Intranasal dexmedetomidine administration

Upon arrival for the scheduled procedure, a 4 mcg/kg dose (maximum 200 mcg) of intranasal dexmedetomidine (100 mcg/mL), divided into the two nostrils, was administered by the parents with direct supervision by the clinicians or by the clinician if the parents preferred, through a mucosal atomizer device. Although tilting the head back was encouraged during administration, focus was placed on position of comfort, for example in the parent's arms. To augment desensitization, the device was then given to the child to take home.

Communication, clinical environment, and monitoring

© 2023 Sahyoun C et al. *JAMA Network Open.*

The procedure room was kept as quiet as possible with the number of staff kept at a minimum and the lights dimmed. The use of objects or devices such as mobile phones with the child's preferred music or video, guided by parental input, was encouraged. Patient safety monitoring (pulse oximetry, three-lead electrocardiogram, non-invasive blood pressure monitoring) was started only after drug administration, when patients achieved a modified Ramsay Sedation Scale (RSS) of 3 (table 1), to avoid frightening them. After the administration of dexmedetomidine, sleeping conditions were maximized by dimming the lights further and by communicating through whispering. Parents were encouraged to place their child in their usual sleeping position. Whenever the patient and family did not speak the languages spoken by at least one member of the sedation team, an in-person interpreter was arranged in advance.

N_2O administration

Once the patient was asleep, parents supervised by clinicians applied an unscented face mask delivering a free-flowing mixture of 50% N_2O/50%Oxygen. Per institutional guidelines, patients were fasted two hours prior to the start of N_2O administration.

Local anesthesia

When accepted by patients, Eutectic Mixture of Local Anesthetics (EMLA) cream was applied at home by parents, using a pictogram on proper placement at venipuncture and vaccination sites, at least one hour prior to hospital arrival. If the child refused home EMLA placement, once adequately sedated, subcutaneous buffered 1% lidocaine was injected at the site chosen for venipuncture three minutes prior to puncture.

© 2023 Sahyoun C et al. *JAMA Network Open.*

Quality Improvement database metrics

The database included patient demographic and procedure data and the following sedation data: time from IN dexmedetomidine administration to onset of sedation (RSS 5), duration of N_2O administration, time from dexmedetomidine administration to awakening (RSS 2), time from dexmedetomidine administration to readiness for discharge (modified Aldrete recovery score of 9 or 10/10), need for physical restraint, and adverse events during the sedation. Parental satisfaction was measured on a 0 to 5 Likert scale ("how satisfied were you with the management of your child's wellbeing during today's sedation"), as well as the outcome of standardized phone calls to the parents placed on the evening of the sedation and 24 hours after the sedation, inquiring about the child's behavior and adverse effects since returning home (including vomiting, agitation, aggression, and eating and sleeping disturbances).

© 2023 Sahyoun C et al. *JAMA Network Open.*

eTable 1. Modified Ramsay Sedation Scale

1	Awake and alert, minimal or no cognitive impairment
2	Awake but tranquil, purposeful responses to verbal commands at conversational level
3	Appears asleep, purposeful responses to verbal commands at conversational level
4	Appears asleep, purposeful responses to verbal commands but only it at louder than usual conversational level or in response to light glabellar tap
5	Asleep, sluggish purposeful responses only to loud verbal commands or strong glabellar tap
6	Asleep, sluggish purposeful responses only to painful stimuli
7	Asleep, reflex withdrawal to painful stimuli only
8	Unresponsive to external stimuli, including pain

eTable 2. Modified Aldrete Recovery Score

Activity	Able to move 4 extremities<br>Able to move 2 extremities<br>Unable to move extremities	2<br>1<br>0
Respiration	Able to breathe deeply and cough freely<br>Dyspnea or limited breathing<br>Apneic	2<br>1<br>0
Circulation	Blood pressure +/- 20% of pre-anesthetic level<br>Blood pressure +/- 20-49% of pre-anesthetic level<br>Blood pressure +/- 50% of pre-anesthetic level	2<br>1<br>0
Consciousness	Fully awake<br>Arousable on calling<br>Not responding	2<br>1<br>0
Oxygen saturation	Able to maintain O_2 saturation >92% on room air<br>Needs oxygen to maintain O_2 saturation >90%<br>O_2 saturation <90% even with supplemental oxygen	2<br>1<br>0

© 2023 Sahyoun C et al. *JAMA Network Open.*
