# Supplementary material for: Safety and Efficacy Associated With a Family-Centered Procedural Sedation Protocol for Children With Autism Spectrum Disorder or Developmental Delay
Source: JAMA Netw Open. 2023 May 30;6(5):e2315974. doi: 10.1001/jamanetworkopen.2023.15974 (PMC10230313; doi:10.1001/jamanetworkopen.2023.15974)
Supplement: Supplement 2. — Data Sharing Statement [file jamanetwopen-e2315974-s002.pdf]

Data Sharing Statement

Data
Data available: Yes
Data types: Deidentified participant data, Data dictionary
How to access data: cyril.sahyoun@hcuge.ch
When available: With publication

Supporting Documents
Document types: None

Additional Information
Who can access the data: researchers whose proposed use of the data has been approved by an ethics committee
Types of analyses: For the purpose of further research on the topic
Mechanisms of data availability: with a signed data access agreement
